# Ndd1 Turnover by SCF^Grr1^ Is Inhibited by the DNA Damage Checkpoint in *Saccharomyces cerevisiae*

**DOI:** 10.1371/journal.pgen.1005162

**Published:** 2015-04-20

**Authors:** Ellen R. Edenberg, Kevin G. Mark, David P. Toczyski

**Affiliations:** Department of Biochemistry and Biophysics, University of California, San Francisco, San Francisco, California, United States of America; University of Massachusetts Medical School, UNITED STATES

## Abstract

In *Saccharomyces cerevisiae*, Ndd1 is the dedicated transcriptional activator of the mitotic gene cluster, which includes thirty-three genes that encode key mitotic regulators, making Ndd1 a hub for the control of mitosis. Previous work has shown that multiple kinases, including cyclin-dependent kinase (Cdk1), phosphorylate Ndd1 to regulate its activity during the cell cycle. Previously, we showed that Ndd1 was inhibited by phosphorylation in response to DNA damage. Here, we show that Ndd1 is also subject to regulation by protein turnover during the mitotic cell cycle: Ndd1 is unstable during an unperturbed cell cycle, but is strongly stabilized in response to DNA damage. We find that Ndd1 turnover in metaphase requires Cdk1 activity and the ubiquitin ligase SCF^Grr1^. In response to DNA damage, Ndd1 stabilization requires the checkpoint kinases Mec1/Tel1 and Swe1, the *S*. *cerevisiae* homolog of the Wee1 kinase. In both humans and yeast, the checkpoint promotes Wee1-dependent inhibitory phosphorylation of Cdk1 following exposure to DNA damage. While this is critical for checkpoint-induced arrest in most organisms, this is not true in budding yeast, where the function of damage-induced inhibitory phosphorylation is less well understood. We propose that the DNA damage checkpoint stabilizes Ndd1 by inhibiting Cdk1, which we show is required for targeting Ndd1 for destruction.

## Author Summary

All cells must regulate cell division in response to extracellular and intracellular cues, and one of the most critical steps to regulate is the process of cell division, or mitosis. In response to DNA damage in budding yeast, cells activate a checkpoint that promotes DNA repair and arrests the cell cycle before division to give the cell time to repair the lesion. One of the key regulators of mitosis is an essential transcription factor called Ndd1. Ndd1 is known to be regulated by transcription and phosphorylation, both in unperturbed cells and following exposure to DNA damage. Here, we show that Ndd1 protein turnover is also regulated in both situations. Ndd1 is degraded quickly during an unperturbed cell cycle, but is strongly stabilized following exposure to DNA damage. We identify the machinery that targets Ndd1 for turnover and the signaling pathways required to stabilize Ndd1 in response to DNA damage. Maintaining high levels of Ndd1 after exposure to DNA damage may allow the cell to reactivate Ndd1 after the damage has been repaired to promote mitosis.

## Introduction

Ndd1 is the dedicated transcriptional activator of a cluster of genes in *Saccharomyces cerevisiae* called the *CLB2* cluster [1]. This cluster consists of thirty-three members, including the namesake cyclin *CLB2*, as well as other important mitotic regulators, such as the yeast polo kinase *CDC5* and the activator of the anaphase promoting complex, *CDC20* [2]. Transcription of this cluster is regulated during the cell cycle [1–10] and in response to DNA damage [11–14] through regulation of Ndd1.

Ndd1 is regulated both transcriptionally and post-transcriptionally during the mitotic cell cycle in order to control transcription of the *CLB2* cluster. Ndd1 is itself a member of the Hcm1-regulated transcriptional cluster [15] and transcriptionally peaks in early S phase. Genome-wide studies found that the Swi4 and Swi6 transcription factors bound upstream of Ndd1, suggesting that Ndd1 may also be regulated by a G1 transcriptional regulator, the SBF, since [16]. Ndd1 is also regulated post-transcriptionally by phosphorylation. Throughout the cell cycle, Fkh2 and Mcm1 bind to *CLB2* cluster promoters and coordinate both the activation and repression of the cluster by associating with Ndd1 as well as transcriptional repressors [3,4]. Ndd1 binding to Fkh2 in early mitosis requires phosphorylation by Clb2-Cdk1 and Cdc5 (the yeast Polo kinase) [5,7,9]. The phosphorylation of Ndd1 by Clb2-Cdk1 and Cdc5 also promotes the intrinsic transcriptional activator activity of Ndd1 [5,7,9]. Ndd1 activity is further regulated by Protein Kinase C (PKC), which phosphorylates Ndd1 to inhibit its activity early in S phase [6]. Recent work has also shown that Ndd1 protein turnover is regulated during the meiotic cell cycle by the Anaphase Promoting Complex and its meiotic specific substrate adaptor, Ama1 [17].

In response to DNA damage, Ndd1 is inhibited by a signal transduction cascade called the DNA damage checkpoint [11,13,14]. The checkpoint is activated by the kinases Mec1 and Tel1, homologs of human ATR and ATM, respectively. In turn, Mec1 and Tel1 activate the downstream effector kinases, Rad53, Dun1, and Chk1 [18,19]. *RAD53*-dependent Ndd1 phosphorylation inhibits Ndd1 by blocking its recruitment to Fkh2-bound promoters, leading to the transcriptional down-regulation of the *CLB2* cluster [11,13,14]. Here, we characterize Ndd1 turnover during the mitotic cell cycle and in response to DNA damage. We find that Ndd1 turns over quickly in an unperturbed metaphase, in a Cdk1- and SCF^Grr1^-dependent manner. In addition, we find that Ndd1 is stabilized by Mec1/Tel1 in response to DNA damage through inhibitory phosphorylation of Cdk1. Thus, the DNA damage checkpoint simultaneously inhibits Ndd1 function by phosphorylation, and also preserves the inhibited form. We speculate that the stabilized, inhibited Ndd1 may allow the cell to remain primed to undertake mitotic transcription once the checkpoint is inactivated.

## Results

### Ndd1 turns over in the cell cycle and is stable in response to DNA damage

**Fig 1:**
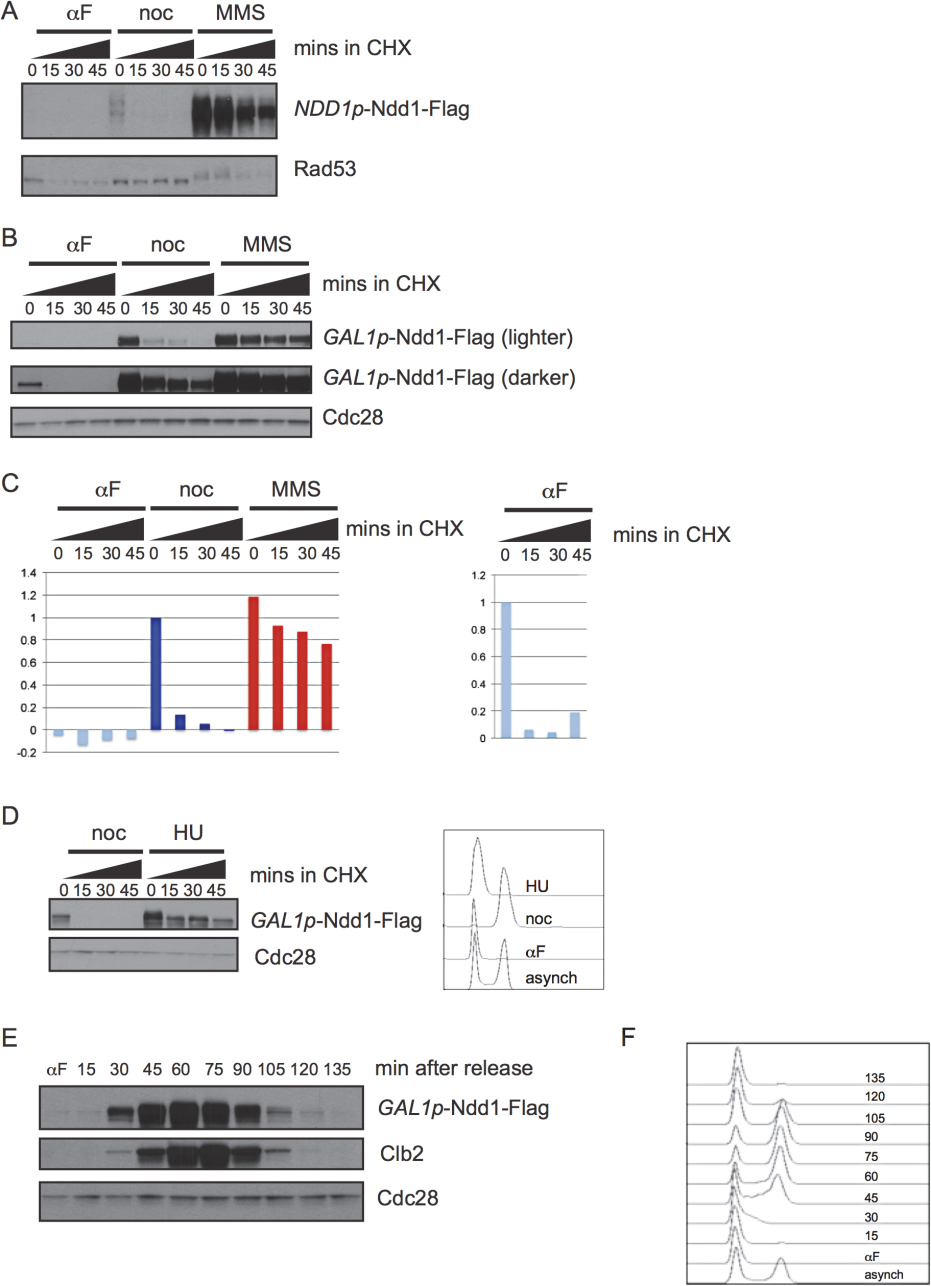
Regulation of Ndd1 turnover. A) Cells expressing Ndd1-Flag from its endogenous promoter were arrested in G1 (with 10 μg/ml α–g/mlh (αF)), metaphase (with 10 μg/ml nocodazole (noc)), or exposed to DNA damage (with 0.05% MMS) for 2.5 hours. Cycloheximide (CHX) was added at 50 μg/ml at t = 0 and protein turnover was followed with 15 minute timepoints. Rad53 is shown as a control for damage treatment, because it hyper-shifts in response to DNA damage. B) Expression of Ndd1-Flag from the *GAL1* promoter (*GAL1*p-Ndd1-Flag) was induced by growth in galactose for 6.5 hours until cells reached mid-log phase. Cells were treated with αF, noc, or MMS for 2.5 hours and turnover was followed as in [A]. Cdc28 is shown as a loading control. C) Quantification is shown of samples in [B]. On the left, quantification is shown from lighter exposure. Right panel shows quantification of αF samples only from the darker exposure. Y-axis shows the normalized signal above background normalized to the value at t = 0 in nocodazole arrested cells (left) or αF arrested cells (right). D) Cells expressing *GAL1*p-Ndd1-Flag were induced by growth in galactose for 3 hours, then arrested in G1 with 10 μg/ml αF for 2.5 hours, and released into media containing either 10 μg/ml nocodazole or 0.2 M hydroxyurea (HU). Cycloheximide was added after 1.5 hours and protein turnover was followed with 15 minute timepoints. FACS analysis shows G1 peak from the αF arrest and cell cycle progression following 1.5 hours release into HU and noc. E) *GAL1*p-Ndd1-Flag was induced with galactose until cells reached mid-log phase. Cells were arrested in G1 with αF for 2.5 hours, and released into the cell cycle and Ndd1 was examined. αF was re-added to the media after the 30 minute timepoint was collected to prevent the cells from entering a second cell cycle. Clb2 levels are shown to indicate cell cycle position. Cdc28 is shown as a loading control. F) FACS analysis showing cell cycle progression of timepoints shown in [F].

We first asked whether Ndd1 protein turnover was regulated over a normal cell cycle. To examine protein turnover, we inhibited new protein synthesis with cycloheximide and followed the degradation of the protein over time. We found that Ndd1 was unstable in metaphase-arrested cells; however, Ndd1 was stable following treatment with the DNA damaging agent methyl methane sulfonate (MMS) (Fig 1A). Under its endogenous cell cycle-regulated promoter, Ndd1 protein was undetectable in G1-arrested cells [1,15]. In order to study Ndd1 turnover in the absence of its transcriptional regulation, we placed a Flag-tagged, second copy of *NDD1* under the control of the *GAL1* promoter. As we had seen with Ndd1 under its endogenous promoter, *GAL1*p-Ndd1 was unstable in metaphase-arrested cells and stabilized following treatment with MMS (Fig 1B, also shown in S1 Fig with FACS for cell cycle progression). Ndd1 was even more unstable in G1-arrested cells than in metaphase-arrested cells (Fig 1B), as seen by both its rate of turnover and its lower starting level (Fig 1B and quantified in Fig 1C). We next wondered whether the stabilization of Ndd1 was also induced by DNA replication checkpoint signaling. In order to test this, we arrested cells in G1 using α-factor and released them into the presence of nocodazole or hydroxyurea (HU) for 1.5 hours. Similar to what we observed with MMS treatment, cells exposed to HU treatment strongly stabilized Ndd1, while Ndd1 turned over rapidly in nocodazole treated cells (Fig 1D with FACS showing arrest; quantification shown in S2A Fig).

To determine whether the cell cycle regulated turnover we observed was sufficient to generate periodic expression of Ndd1 in the absence of transcriptional regulation, we arrested cells expressing *GAL1*p-Ndd1 in G1 with α-factor, released the cells into fresh media to allow them to enter the cell cycle, and followed Ndd1 levels into the next G1. Ndd1 protein levels were very low in G1, rose through the cell cycle, and dropped in the next G1 (Fig 1E, with FACS analysis of cell cycle progression shown in Fig 1F). This result confirms that protein turnover of Ndd1 is regulated during the cell cycle, as had previously been suggested [1]. Furthermore, it suggests that the changes in protein turnover rate during the cell cycle are sufficient for periodic Ndd1 expression in the absence of Ndd1 transcriptional regulation.

### Cdk1 kinase promotes Ndd1 turnover

**Fig 2:**
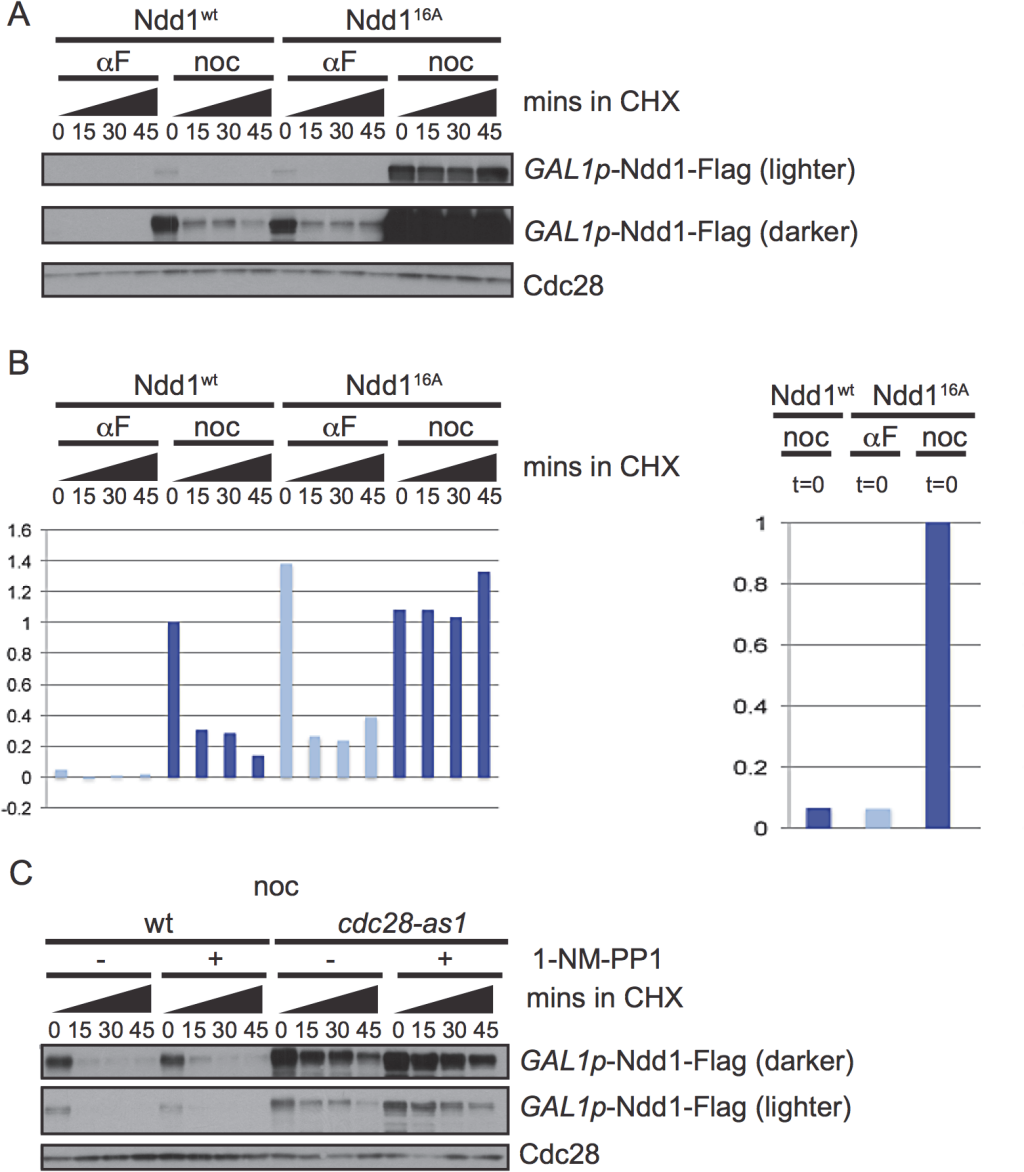
Ndd1 turnover requires Cdk1, Hog1, and three of the GSK3 family kinases. A) Cells expressing wildtype Ndd1-Flag (*GAL1*p-Ndd1^wt^-Flag) or an allele with 16 S/T-P consensus sites mutated (*GAL1*p-Ndd1^16A^-Flag) were induced with galactose, arrested with αF or noc for 2.5 hours and examined after cycloheximide (CHX) addition as in Fig 1B. Cdc28 is shown as a loading control. B) Quantification of the experiment in [A] is shown. On the left is the quantification from the darker exposure, while right side shows relative abundance quantified from the lighter exposure. Y-axis shows the normalized signal above background normalized to the value at t = 0 in nocodazole arrested Ndd1^wt^ cells (left) or Ndd1^16A^ cells (right). C) *GAL1p*-Ndd1^wt^-Flag was induced with galactose in a wildtype or *cdc28-as1* strain. Cells were then arrested in metaphase with nocodazole for 3.5 hours, with treatment of 1 μM 1-NM-PP1 for the last hour, as noted. Cycloheximide was then added at t = 0 and turnover was followed as in [A]. Cdc28 is shown as a loading control.

Exposure to DNA damaging agents leads to budding yeast cells arresting in metaphase; however Ndd1 turnover is strikingly different between nocodazole-arrested cells (which arrest in metaphase in the absence of DNA damage) and cells exposed to MMS. Ndd1 is phosphorylated by many kinases during the cell cycle [5–7,9]. Since phosphorylation is a common mode of regulating substrate turnover, we hypothesized that Ndd1 phosphorylation may regulate its turnover. First, we tested whether the 16 S/T-P (serine or threonine, followed by a proline) sites on Ndd1 activated its turnover in metaphase. These sites are the minimal consensus motif for CDK and MAP kinases. Clb2-Cdk1 is known to phosphorylate Ndd1 in order to promote its activity [7,9]. Because the Cdk1 phosphorylation of Ndd1 is essential for its function [7,9], we placed a Flag-tagged, second copy of *NDD1* ^*16A*^ under the control of the *GAL1* promoter. Ndd1^16A^ was completely stabilized in metaphase-arrested cells, consistent with phosphorylation by Cdk1 driving turnover during mitosis (Fig 2A, quantification shown in Fig 2B). However, Ndd1 was even more unstable in α-factor-arrested cells, and while Ndd1^16A^ was stabilized compared to Ndd1^wt^ in G1, it was still quite unstable (Fig 2A), suggesting that additional kinases might phosphorylate Ndd1 in these arrested cells. In order to test whether Cdk1 itself was required for Ndd1 turnover in metaphase, we used a strain in which the yeast Cdk1 (*CDC28*) has been replaced with an analog sensitive allele, *cdc28-as1*. This allele is sensitive to inhibition by the ATP analog 1-NM-PP1, but is also hypomorphic, with a 6-fold lower k_cat_, even in the absence of the inhibitor [20]. We arrested cells in metaphase using nocodazole, then treated with 1-NM-PP1 (or held the cells in nocodazole without the inhibitor), and followed turnover of Ndd1 for 45 minutes following the addition of cycloheximide. Wildtype cells were unaffected by treatment with 1-NM-PP1, and Ndd1 had the expected short half-life (Fig 2C, quantification shown in S2B Fig). Consistent with the *cdc28-as1* allele being hypomorphic [20], Ndd1 was significantly stabilized in *cdc28-as1* cells even in the absence of the inhibitor. Moreover, this suggests that the phosphorylation of Ndd1 that promotes its turnover is very sensitive even to small changes in CDK activity. Treatment with 1-NM-PP1 in these cells led to a greater stabilization of Ndd1. Based on these data, we propose that CDK is required for Ndd1 turnover in metaphase.

### Ndd1 is turned over by SCF^Grr1^

**Fig 3:**
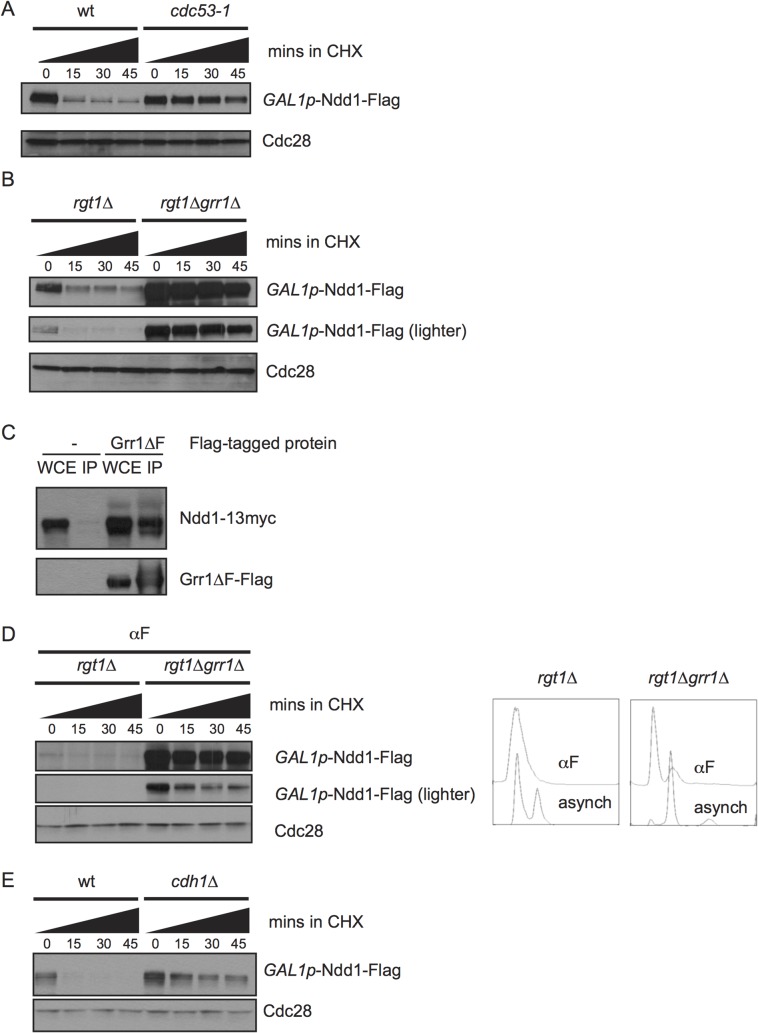
SCF^Grr1^ turns over Ndd1. A) *GAL1p*-Ndd1-Flag was induced with galactose in wildtype or *cdc53-1* strains at 23°C for 5 hours. All strains were then shifted to 37°C for 2.5 hours to inactivate the temperature-sensitive allele before addition of cycloheximide (CHX). Turnover was then examined as in Fig 1B, except at 37°C. Cdc28 is shown as a loading control. B) *GAL1p*-Ndd1-Flag was induced with galactose in *rgt1Δ* or *rgt1Δ grr1Δ* strains for 2.5 hours. Protein turnover was followed as in [A]. C) Strains expressing *NDD1p*-Ndd1-13Myc with either an empty vector (pRS426) or pYES-Grr1*Δ*F-Flag (containing a galactose-inducible copy of Grr1-Flag lacking its F-box domain) were grown in galactose media overnight to double twice. Whole cell extracts (WCE) were prepared and immunoprecipitated with anti-Flag antibody, Western blotted and probed for Flag and Myc. D) Experiment was performed as in [B], expect that cells were pre-arrested in G1 using 10 μg/ml α–/ml im (αF) for 6 hours. *grr1Δ* cells do not arrest well, but FACS analysis shows strong enrichment of G1 peak. E) Experiment was performed as in [B] in wt or *cdh1Δ* strains.

We next wanted to know which E3 ligase targeted Ndd1 for degradation. Each member of the SCF family of E3 ligases contains the cullin Cdc53, Skp1, and the Ring finger subunit Rbx1, in addition to any one of several substrate adaptors called F-box proteins [21]. Budding yeast have 20 putative F-box proteins, most of which are thought to target a specific suite of substrates [22]. We found that Ndd1 was strongly stabilized at the non-permissive temperature in the temperature-sensitive mutant *cdc53-1* (Fig 3A, quantification shown in S2C Fig), suggesting that Ndd1 is a substrate of an SCF ligase.

The F-box protein Grr1 targets several cell cycle and metabolic proteins for turnover, including the regulator of glycolysis Pfk27 and the G1 cyclins [22–25]. *grr1Δ* strains grow very slowly [26,27], and simultaneous deletion of *RGT1*, a glucose-responsive transcription factor, partially rescues this growth defect [28]. Therefore, to examine the effect of *GRR1* deletion on Ndd1 stability, we compared Ndd1 turnover in *rgt1Δ* cells and *grr1Δ rgt1Δ* cells. We found that Ndd1 was strongly stabilized by deletion of *GRR1* (Fig 3B, quantification shown in S2D Fig), which suggests that Grr1 is the primary F-box protein that targets Ndd1 for turnover. In order to test whether Ndd1 was a direct substrate of Grr1, we tested whether Ndd1 physically bound Grr1. We used an allele of Grr1 lacking its F-box (Grr1*Δ* F), such that it could not interact with the rest of the SCF, but was still able to bind substrates. This allele allowed us to overexpress Grr1 without promoting substrate turnover [22,23]. Grr1*Δ* F-Flag was immunoprecipitated from cells, and extracts were probed for Ndd1-myc. As shown in Fig 3C, Ndd1-myc is specifically pulled down with Grr1*Δ* F-Flag, suggesting that Ndd1 is a direct substrate of Grr1. Grr1 interacts with its known substrates by binding to negatively charged phosphorylation on the substrates through Grr1’s positively charged leucine rich repeats, but there is no known consensus site for the Grr1 phosphodegron [29]. We found that Grr1 also contributed to the very fast turnover of Ndd1 in G1-arrested cells (Fig 3D, quantification shown in S2E Fig), suggesting that Cdk1-dependent phosphorylation of Ndd1 cannot be the only mechanism by which Grr1 recognizes Ndd1 as its substrate. In addition, we tested whether the Anaphase Promoting Complex (APC) was involved in Ndd1 turnover. Cdh1 is the APC adaptor protein active during G1. In *cdh1Δ* cells, Ndd1 is also stabilized (Fig 3E, quantification shown in S2F Fig), suggesting that APC^Cdh1^ may also contribute to the very quick turnover of Ndd1 observed in G1.

### Ndd1 stability in response to DNA damage requires Swe1

**Fig 4:**
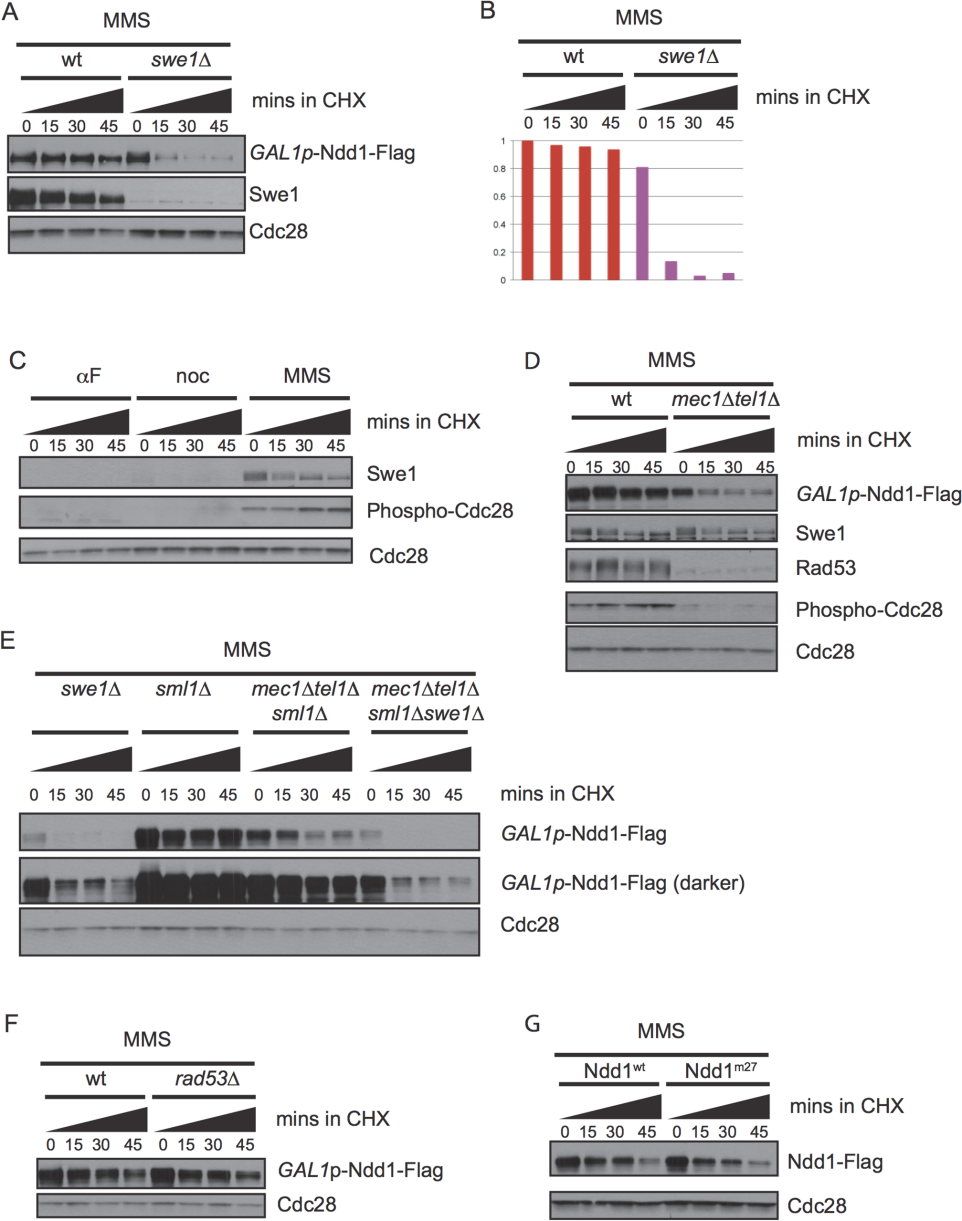
Ndd1 stabilization in response to DNA damage requires Swe1 and Mec1/Tel1. A) *GAL1p*-Ndd1-Flag was induced with galactose for 2.5 hours in wildtype or *swe1Δ* strains. Cells were then exposed to MMS for 2.5 hours in galactose. Cycloheximide (CHX) was added at t = 0, samples were taken every 15 minutes, blotted and probed for Swe1, Flag, and total Cdc28, as a loading control. B) Quantification of experiment shown in [A]. Y-axis shows the normalized signal above background normalized to the value at t = 0 in wildtype cells. C) Samples from Fig 1B were re-run and probed for Swe1 and Phospho-Y19 Cdc28 to follow Swe1 turnover in αF, noc, or MMS. Cdc28 loading control is reproduced from Fig 1B. D) *GAL1p*-Ndd1-Flag was induced with the addition of galactose in wildtype or *mec1Δ tel1Δ* strains. Cells were then exposed to 0.05% MMS for 4.5 hours. CHX was added at t = 0 and turnover was followed as in [A]. Rad53 is shown as a control for *mec1Δ tel1Δ*. Swe1 and Phospho-Y19 Cdc28 shown are from the same samples but run on a separate gel. Cdc28 is shown as a loading control. E) Experiment was performed as in [D] using *swe1Δ*, wt, *mec1Δtel1Δ*, *swe1Δmec1Δtel1Δ* strains. All *mec1Δtel1Δ* strains were rescued by simultaneous deletion of *sml1Δ*. F) Experiment was performed as in [D] with *sml1Δ* and *rad53Δsml1Δ* strains. G) Ndd1^wt^-Flag and Ndd1^m27^-Flag under the endogenous Ndd1 promoter were grown to mid-log phase (3–4 hours) and then treated with MMS for 2.5 hours. Cycloheximide was added at t = 0 and turnover was followed for 45 minutes with 15 minute timepoints taken as in [D].

Our finding that Ndd1 was strongly stabilized in cells treated with the DNA damaging agent MMS (Fig 1A and Fig 1B) provided an explanation for previous work that had found Ndd1 was expressed at high levels in damaged-arrested cells [14,30,31]. Since Ndd1 turnover is mediated by Cdk1 activity, we examined whether Ndd1 stabilization in response to DNA damage required the kinase Swe1 (Fig 4A, quantified in Fig 4B). Swe1 is the *S*. *cerevisiae* homolog of Wee1, which inhibits Cdk1 by phosphorylation on a conserved tyrosine residue [32]. Wee1 homologs in other organisms are important for cell cycle arrest in response to DNA damage, but Swe1 is dispensable for this arrest in budding yeast [33,34]. Nonetheless, both Swe1 and its downstream inhibitory phosphorylation on Cdk1 accumulate in response to DNA damage [33,34], although there have been conflicting reports in the literature of the involvement of the damage checkpoint signaling pathway on the accumulation of Swe1 [30,35,36]. We find that both Swe1 and its downstream inhibitory phosphorylation on Cdk1 are present at high levels in cells arrested with MMS compared to cells arrested in metaphase with nocodazole (Fig 4C). Swe1 phosphorylation specifically inhibits Clb2-Cdk1 kinase function [37]. While Swe1 and the inhibitory phosphorylation of Cdk1 are dispensable for cell cycle arrest in response to DNA damage, Clb2 activity is partially inhibited [33,34]. Consistently, Cdk phosphorylation of Ndd1 has been shown to be mediated by Clb2-Cdk1 [7,38], suggesting that the checkpoint-mediated block to Ndd1 turnover is through Clb2/Cdk inhibition by Swe1 (see Discussion for more detail).

We also found that stabilization of Ndd1 required the DNA damage checkpoint, as *mec1Δtel1Δ* mutants failed to stabilize Ndd1 after exposure to MMS (Fig 4D, quantification shown in S2G Fig). We found that Swe1 was stable independent of Mec1 and Tel1, consistent with an earlier report that Swe1 stability in response to DNA damage and replication stress was independent of Mec1 and its downstream kinase Rad53 [36]. We do, however, observe less Cdc28 inhibitory phosphorylation in the *mec1Δtel1Δ* mutants. A previous mass spectrometry study identifying proteome-wide phosphorylations in budding yeast treated with MMS did identify a phosphorylated residue on the phosphatase Mih1 that removes the Cdk1 inhibitory phosphorylation [39]. Though neither the signaling pathways upstream of the phosphorylation of Mih1 nor the downstream effects have been characterized, this result suggests that Mec1 and Tel1 could modulate Cdk1 signaling by affected Mih1. However, since Swe1 is required for the Cdk1-inhibitory phosphorylation, we found that the destabilization of Ndd1 observed in *swe1Δ* mutants was epistatic to that observed in *mec1Δtel1Δ* mutants (Fig 4E, quantification shown in S2H Fig).

Previously, our lab and others have identified Rad53-dependent phosphorylation of Ndd1 that inhibits its function in response to DNA damage signaling [11,13,14]. We therefore tested whether the stabilization of Ndd1 in response to DNA damage was controlled by the same pathway. Surprisingly, we found that Ndd1 stabilization is independent of both Rad53 (Fig 4F, quantification shown in S2I Fig) and the 27 Rad53-dependent phosphorylation sites that inhibit its function in response to DNA damage (Fig 4G, quantification shown in S2J Fig), suggesting that two opposing methods of regulation (inhibition and stabilization) independently converge on Ndd1 in response to DNA damage.

## Discussion

**Fig 5:**
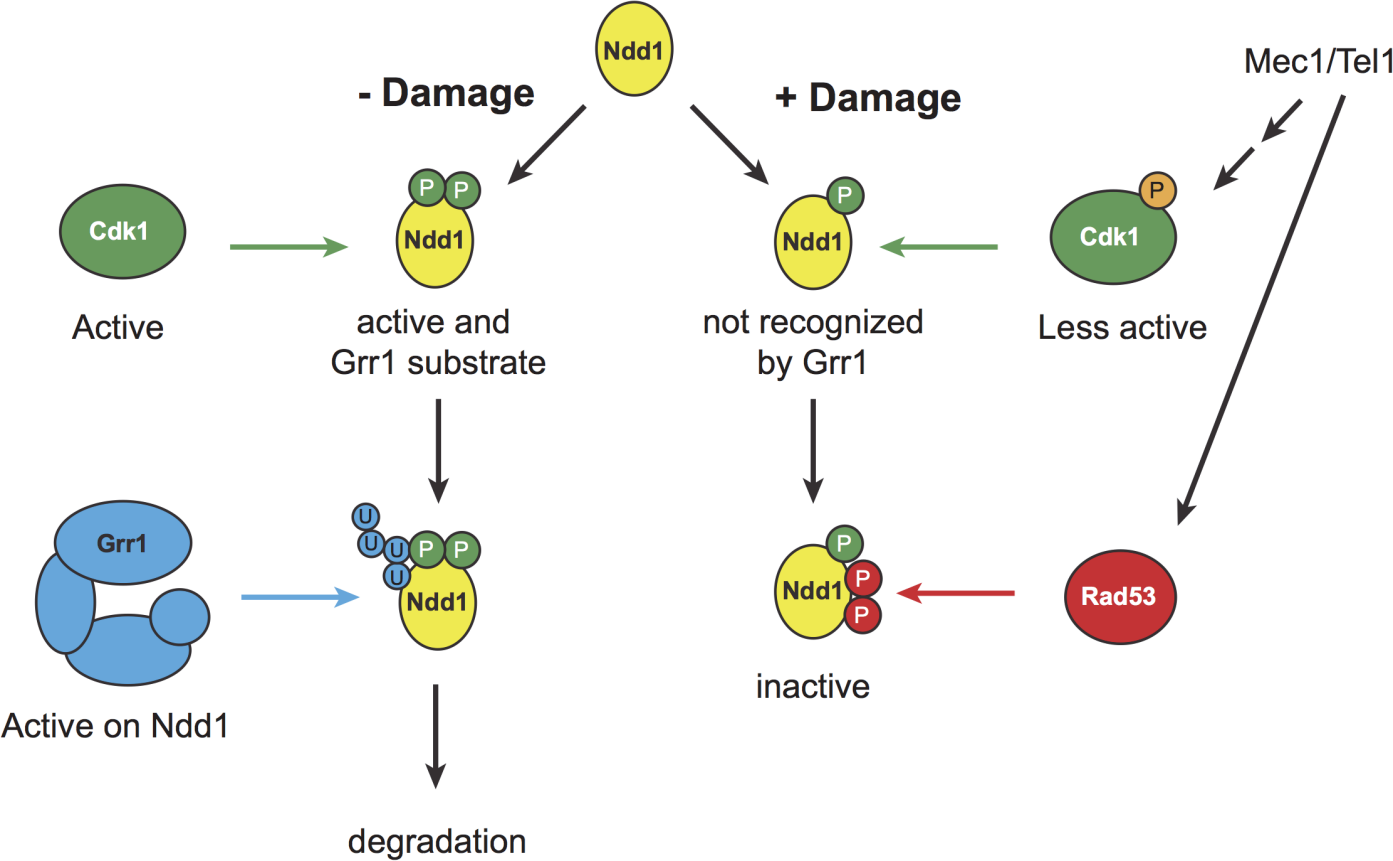
Model for mitotic turnover of Ndd1. In the absence of DNA damage, Ndd1 is phosphorylated by Cdk1, which promotes Ndd1 transcriptional activity but also enables SCF^Grr1^ to recognize Ndd1 as a substrate. Ndd1 is therefore ubiquitinated and subsequently degraded. In response to DNA damage, Swe1 levels are increased, and Cdk1 is inhibited by phosphorylation which lowers its activity several fold. Ndd1 is no longer recognized by Grr1 and is therefore stabilized. Mec1 and Tel1 are important for maintaining high levels of phospho-Cdk1, and therefore help to stabilize Ndd1, either by promoting Swe1 activity or by inhibiting the phosphatase Mih1 (homolog of Cdc25). While Ndd1 is stabilized, its activity is independently inhibited by Rad53-dependent phosphorylation, leading to the accumulation of inhibited Ndd1. We speculate that maintaining this inhibited pool of Ndd1 may allow the cell to rapidly re-enter the cell cycle once the damage is repaired and the checkpoint is turned off.

In this work, we show that Ndd1 protein turnover is regulated during the cell cycle and in response to DNA damage. During G1, Ndd1 turnover reinforces the transcriptional regulation of Ndd1 to keep Ndd1 levels low. During metaphase, Ndd1 turnover requires Cdk1 activity (Fig 2C). We find that Ndd1 is stabilized in response to DNA damage (Fig 1A–1C), a result that explains several older observations that Ndd1 protein accumulated in response to DNA damage [14,30,31]. In response to DNA damage, Ndd1 is inhibited by phosphorylation [11,13,14], however the stabilization of Ndd1 we observe is independent of the known signaling and phosphorylation sites that we previously identified (Fig 4F and 4G). We propose a model in which Ndd1 is stabilized in damage, while its activity is independently inhibited (see Fig 5). Our speculation is that accumulated Ndd1 can be quickly de-phosphorylated during recovery following the repair of DNA damage to allow for rapid activation of its transcriptional targets to promote mitosis. Unfortunately, it is difficult to experimentally test this model, due to the fact that CDK phosphorylations promote both activation of Ndd1 and its destruction.

During metaphase, Cdk1 is required for Ndd1 activity. Phosphorylation of Ndd1 by Cdk1 is required both for its recruitment to target gene promoters, by generating a binding site for Fkh2, and for Ndd1’s intrinsic transactivator activity [7,9]. Here, we find that Cdk1 activity is also required to turn over Ndd1 in metaphase (Fig 2C). Recent work on a different cell cycle transcription factor, Hcm1, found that Cdk1 similarly promoted both the activity and the turnover of Hcm1 [40]. This suggests that dual, opposing regulation by Cdk1 may be a common theme in cell cycle regulation. Ndd1 has 16 potential Cdk1 sites that fit its minimal consensus sequence. A subset of these sites has been shown to be essential [9], and therefore presumably required for Ndd1 activation; however, it is still unknown whether other Cdk1 sites on Ndd1 are also essential or important for activation, and whether turnover of Ndd1 and activation of Ndd1 involve the same Cdk1 phosphorylation sites.

In other organisms, Cdk1 inhibitory phosphorylation by Wee1 is an important component of the cellular response to DNA damage and replication stress, with the checkpoint ultimately leading to high levels of Cdk1 phosphorylation to inhibit its function and block the G2/M transition [41–45]. In budding yeast, Cdk1 inhibitory phosphorylation is not required for the checkpoint to arrest cells in response to DNA damage [33,34]. Instead, Swe1 responds to cell wall stress through the morphogenesis checkpoint [46]. In addition, DNA damage has been shown to affect Swe1, although there have been conflicting reports on the effect of DNA damage or replication stress on Swe1 levels. One group found that Swe1 was stabilized in a *MEC1*- and *RAD53*-independent manner [36]. Others have shown that Swe1 levels drop in response to prolonged HU treatment (which leads to replication stress by depleting nucleotides), in a checkpoint-dependent manner [35]. In addition, Swe1 has been shown to transcriptionally accumulate in response to DNA damage, in a Rad53-dependent manner [30]. Here, we find that our 2.5 hour treatment with MMS leads to high levels of Swe1, which is stable in our cycloheximide chase assays (Fig 4C). In addition, we find levels of Swe1 are high independent of DNA damage checkpoint signaling (Fig 4D). We find that DNA damage checkpoint signaling through Mec1 and Tel1 is required for the accumulation of high levels of phosphorylated Cdk1. Mec1 and Tel1 may modify Swe1 activity or may affect Cdk1 inhibition through modulation of the Mih1 phosphatase. In support of this idea, a phosphorylated residue on Mih1 was identified in a proteome-wide study from yeast treated with MMS, but the signaling pathways and the functional consequences have not been studied [39]. Different treatments (either with different drugs to induce damage or replication stress) or different timeframes could explain the observed differences in checkpoint signaling requirements for Swe1 and Cdk1 inhibitory phosphorylation.

Though Swe1 and the inhibitory phosphorylation of Cdk1 are not required for cell cycle arrest in response to DNA damage, our data (Fig 4C) and others [33,34] do find that Cdk1 inhibitory phosphorylation accumulates in response to DNA damage. While many groups have found that Cdk1 kinase is still active in response to DNA damage, Clb2-Cdk1 activity towards H1 is lowered 2- to 4-fold relative to mitotic cells [33,34,47]. Phosphorylated Cdk1 specifically inhibits Clb2-dependent kinase activity [37], and, consistently, Ndd1 is a Clb2-Cdk1 substrate [7,38]. Furthermore, our results using the hypomorphic *cdc28-as1* allele, which has 6-fold lower catalytic activity and significantly stabilizes Ndd1 even in the absence of the 1-NM-PP1 inhibitor, suggests that even modest decreases in Cdk1 activity strongly affect Ndd1 turnover. Even with the 6-fold k_cat_ change in *cdc28-as1*, the doubling time of these cells is not particularly slow (140 minutes v 120 minutes), suggesting that for many of its functions, this level of activity must be sufficient [20]. Therefore, we propose that the two- to four-fold change in kinase activity in response to DNA damage may not affect the phosphorylation of many substrates; perhaps there are other residues in unfavorable positions that are very sensitive to even small changes in Cdk1 activity. Regardless, our results illustrate one of the first significant functions of Cdk1 inhibition in response to DNA damage.

Because Ndd1 is such an important regulator of mitosis through its regulation of transcription of the *CLB2* cluster, many different kinases regulate Ndd1 activity during the cell cycle and in response to stresses such as DNA damage [5–7,9,11,13,14,38]. Here, we find that Ndd1 protein turnover is also controlled during the cell cycle and in response to DNA damage. Moreover, the regulation of Ndd1 protein turnover is independent of the Rad53-dependent inhibition of Ndd1 in response to DNA damage (Fig 4F and 4G). Ndd1 may function as an integration point for many different signals, allowing the cell to ultimately control mitosis.

## Materials and Methods

### Yeast methods

Yeast strains were grown in YM-1 media with 2% dextrose at 30°C unless otherwise noted. Strains were made using standard techniques. Expression from the *GAL1* promoter was induced with 2% galactose. For experiments with temperature-sensitive strains, cells were maintained at 23°C until the experiment.

### Cell cycle and damage treatments

For cell cycle experiments, cells were arrested with 10 μg/ml αg/ml ll μg/ml nocodazole, or 0.05% MMS for 2.5 hours or as noted. For 1-NM-PP1 inhibition of *cdc28as-1*, cells were arrested in nocodazole for 2.5 hours, then held in nocodazole or additionally treated with 1 μM 1-NM-PP1 for 1 more hour.

For FACS analysis of cell cycle progression, cells were fixed in 70% ethanol and stored at 4°C until analysis. Cells were sonicated and treated with 0.25 mg/ml RNase A for 1 hour at 50°C and then digested with 0.125 mg/ml Proteinase K for 1 hour at 50°C. Cells were then labeled with 1 μM Sytox green. Data were collected on a FACSCalibur machine and analyzed with FlowJo software.

### Western blots

From cultures in mid-log phase, cell pellets of equivalent optical densities were collected, washed with 1 ml 4°C H_2_O, and frozen on dry ice. Pellets were thawed in boiling sample buffer (50 mM Tris pH 7.5, 5% SDS, 5 mM EDTA, 10% glycerol, 0.5% β-mercaptoethanol, bromophenol blue, 1 μg/ml leupeptin, 1 μg/ml bestatin, 0.1 mM benzamidine, 1 μg/ml pepstatin A, 5 mM NaF, 1 mM Na_3_VO_4_, 80 mM β-glycerophosphate, 1 mM phenylmethylsulfonyl fluoride). Cells were boiled for 3 minutes, bead-beaten with glass beads for 3 minutes, and clarified by centrifugation. Extracts were analyzed by SDS-PAGE and Western blotting. Western blots were performed with low-salt phosphate buffered saline with Tween-20 (PBS-T) (15 mM NaCl, 1.3 mM NaH_2_PO_4_, 5.4 mM Na_2_HPO_4_, 0.05% Tween-20). Primary antibody incubations were performed in 5% nonfat dry milk and low salt PBS-T. Antibodies were used as follows: α-ilk and low salt PBS-T. Antibodies were used as follows:α-ilk and low salt PBS-T. Antibodies were used asαilk and low salt PBS-T. Antibodies were used as for α-Myc (9E10), α-Myc (9E10), saltD. Kellogg), and α-phospho-Cdc2 (Y15) (antibody was raised against human Cdc2 but recognizes *S*. *cerevisiaie* Cdc28 inhibitory phosphorylation as well, from Cell Signaling Technology #9111).

### Half-life assays

Cells were grown as for Western blotting to mid-log. Cycloheximide was added to cultures for a final concentration of 50 μg/ml after collection of the t = 0 timepoint. Equivalent ODs were collected for each time point, and processed for Western blots as described above. For all experiments with Ndd1 under the control of the *GAL1* promoter, cells were grown in galactose until the cells reached mid-log phase, from 2.5 to 8 hours, before the addition of cycloheximide or treatment for cell cycle arrest or with DNA damaging agent. For half-life experiments in arrested cells, cells were treated with αours, benocodazole, or MMS (still in the presence of galactose) for 2.5 hours before addition of cycloheximide. For *cdc53-1* experiment, cells (including wild-type control) were grown in galactose for 5 hours and then shifted to 37°C for 2.5 hours.

To quantify gels, blots were scanned and quantified using Fiji from most informative exposure. Background levels from the same exposure gel were subtracted from each value. Quantified values were then normalized to corresponding Cdc28 loading control.

### Grr1 binding experiment

Experiment was performed as in [22]. Briefly, strains carrying either pRS426 or pYES2-GRR1ΔF-FLAG-URA3 plasmids were grown in synthetic media lacking uracil with 2% raffinose. Grr1ΔF-Flag was induced with 2% galactose and cultures were allowed to double twice. Cells were lysed in a buffer containing 100 mM Tris-HCl (pH 7.5), 300 mM NaCl, 2 mM EDTA, 0.2% NP-40 with a Roche Complete protease inhibitor tablet without EDTA (one tablet/25 ml), 1 mM PMSF, and four Roche PhosSTOP Phosphatase Inhibitor Tablets (four tablets/25 ml). Lysis was carried out by bead beating, and cleared by centrifugation at 4°C. Lysates were incubated with a 25 μl slurry of anti-Flag M2 Magnetic Beads (Sigma-Aldrich) overnight while rotating at 4°C. Beads were washed three times with PBS buffer containing 0.1% NP-40. Proteins were eluted by mild vortexing in PBS buffer containing 0.1% NP-40 and 500 ng/ml 3X FLAG peptide (Sigma-Aldrich) for 45 min at room temperature. Samples shown are 0.2% of the total input and 20% of the Flag elution.

## Supporting Information

S1 Fig Cell cycle progression shown for nocodazole and MMS treatments. A) Experiment was done as in Fig 1B. Briefly, Ndd1 was expressed from the *GAL1* promoter, cells were treated for 2.5 hours in nocodazole (10 μg/ml) or MMS (0.05%). Cycloheximide was added at t = 0 and protein turnover was followed for 45 minutes. Right side shows the cell cycle progression in these cells. B) Quantification of experiment shown. Y-axis shows the normalized signal above background normalized to the value at t = 0 in nocodazole arrested cells.
(TIFF)

S2 Fig Quantification and half-life analysis of Ndd1. A) Quantification shown from experiment in Fig 1D comparing half-life of cells released from G1 into nocodazole to those released into HU. Y-axis shows the normalized signal above background normalized to the value at t = 0 in nocodazole arrested cells. B) Quantification shown from experiment in Fig 2C, comparing half-life of Ndd1 in the presence and absence of functional Cdc28, normalized to the value at t = 0 in nocodazole arrested cells. C) Quantification is shown from experiment in Fig 3A comparing half-life of Ndd1 in wildtype and *cdc53-1* mutants, normalized to the value at t = 0 in wildtype cells. D) Quantification is shown from experiment in Fig 3B comparing half-life of Ndd1 in *rgt1Δ* and *rgt1Δgrr1Δ* mutants, normalized to the value at t = 0 in *rgt1Δ* cells. Small inset on right shows the quantification from darker exposure of *rgt1Δ* strain only. E) Quantification is shown from experiment in Fig 3D, comparing G1 half-life of Ndd1 in *rgt1Δ* and *rgt1Δgrr1Δ* mutants, normalized to the value at t = 0 in *rgt1Δ* cells. F) Quantification is shown from experiment in Fig 3E, comparing half-life of Ndd1 in wildtype and *cdh1Δ* mutants, normalized to the value at t = 0 in wildtype cells. G) Quantification is shown from Fig 4D, comparing half-life of Ndd1 in wildtype and *mec1Δtel1Δ* mutants, normalized to the value at t = 0 in wildtype cells. H) Quantification is shown from Fig 4E, comparing half-life of Ndd1 and epistasis in wildtype, *swe1Δ* mutants, and *mec1Δtel1Δ* mutants, normalized to the value at t = 0 in *sml1Δ* cells. I) Quantification is shown from Fig 4F, comparing half-life of Ndd1 in wildtype and *rad53Δ* mutants, normalized to the value at t = 0 in wildtype cells. J) Quantification is shown from Fig 4G, comparing half-life of Ndd1^wt^ and Ndd1^m27^, normalized to the value at t = 0 in Ndd1^wt^.
(TIFF)
