# Supplementary figures and images for: Ndd1 Turnover by SCFGrr1 Is Inhibited by the DNA Damage Checkpoint in Saccharomyces cerevisiae
Source: PLoS Genet. 2015 Apr 20;11(4):e1005162. doi: 10.1371/journal.pgen.1005162 (PMC4403921; doi:10.1371/journal.pgen.1005162)

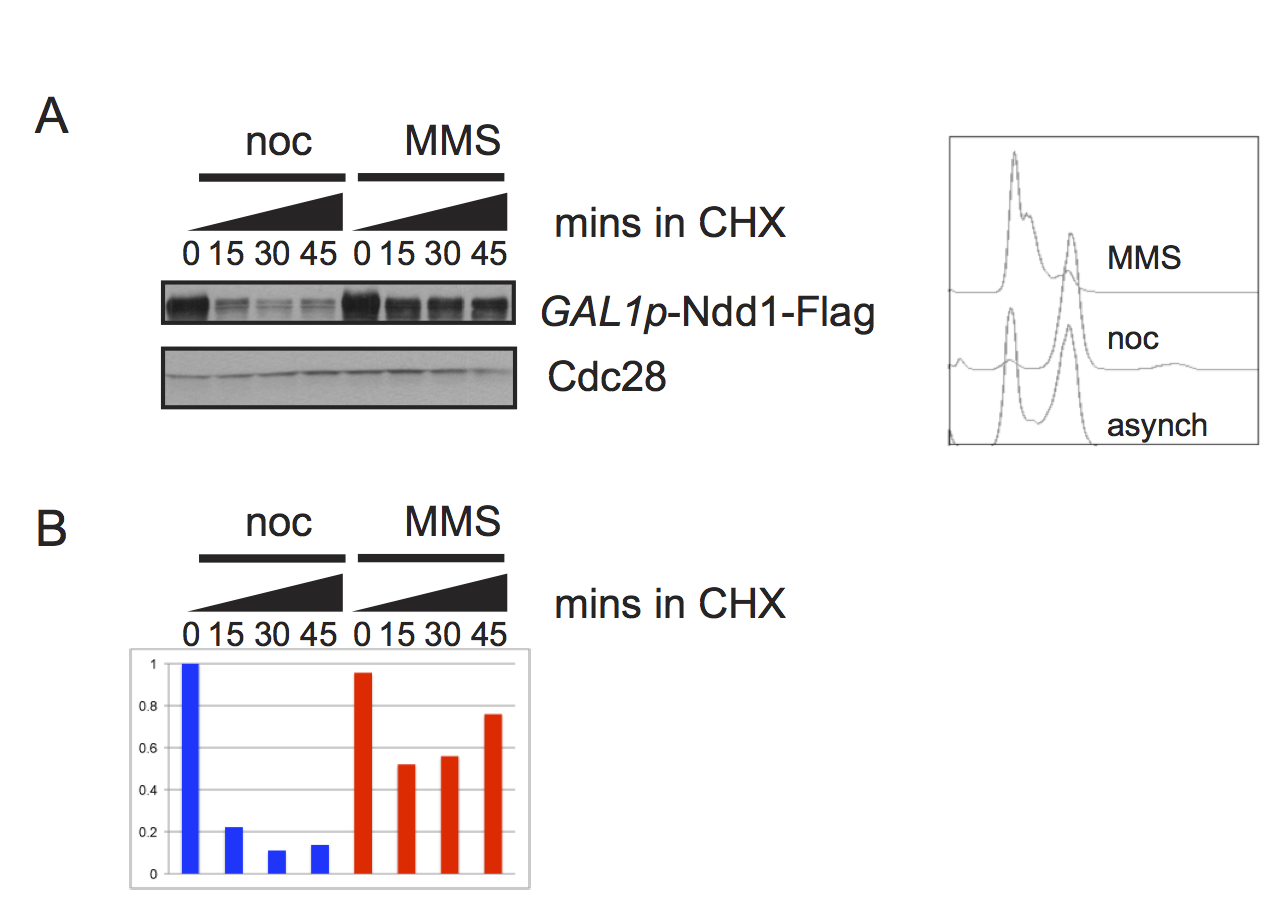

Supplement: S1 Fig — A) Experiment was done as in Fig 1B. Briefly, Ndd1 was expressed from the GAL1 promoter, cells were treated for 2.5 hours in nocodazole (10 μg/ml) or MMS (0.05%). Cycloheximide was added at t = 0 and protein turnover was followed for 45 minutes. Right side shows the cell cycle progression in these cells. B) Quantification of experiment shown. Y-axis shows the normalized signal above background normalized to the value at t = 0 in nocodazole arrested cells. (TIFF) [file pgen.1005162.s001.tiff]

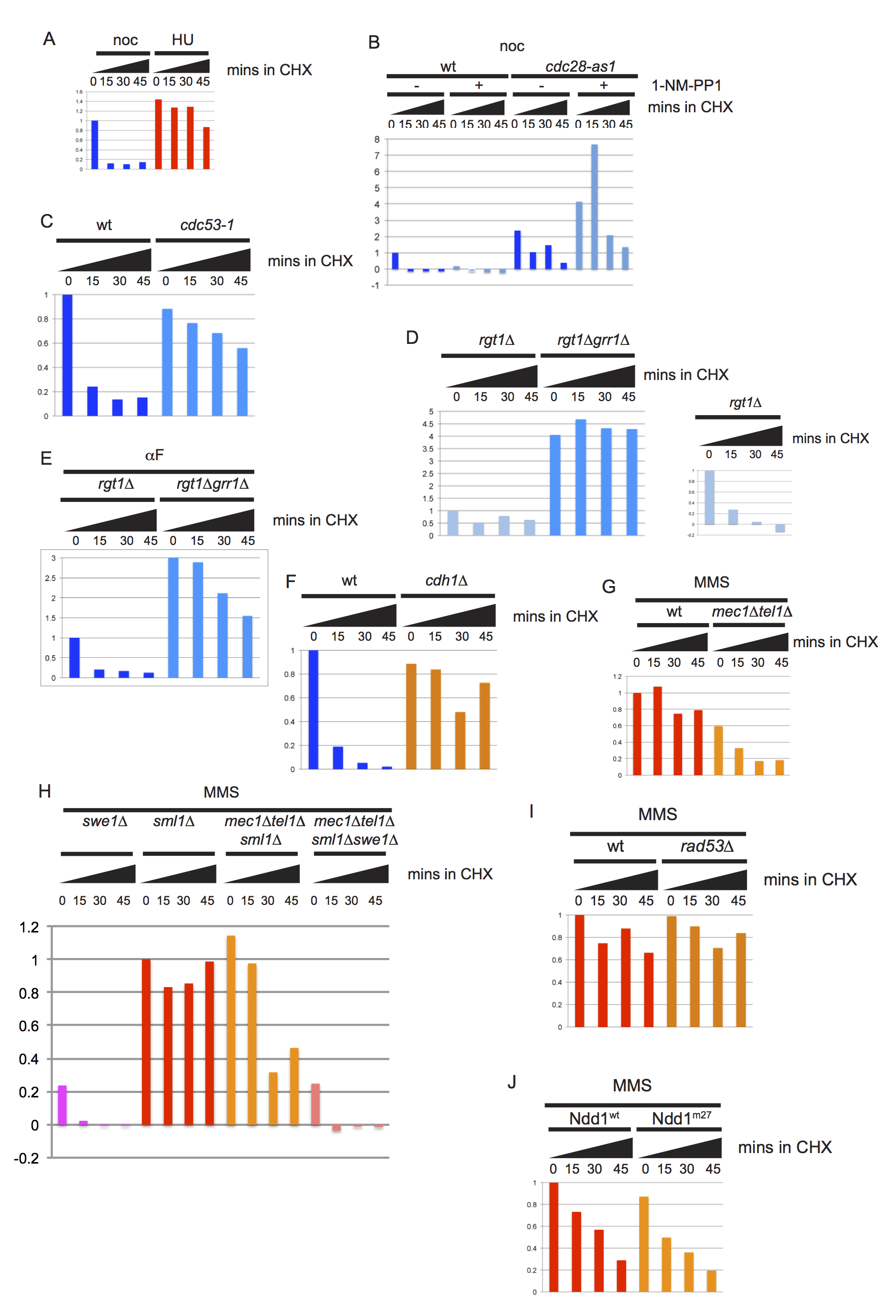

Supplement: S2 Fig — A) Quantification shown from experiment in Fig 1D comparing half-life of cells released from G1 into nocodazole to those released into HU. Y-axis shows the normalized signal above background normalized to the value at t = 0 in nocodazole arrested cells. B) Quantification shown from experiment in Fig 2C, comparing half-life of Ndd1 in the presence and absence of functional Cdc28, normalized to the value at t = 0 in nocodazole arrested cells. C) Quantification is shown from experiment in Fig 3A comparing half-life of Ndd1 in wildtype and cdc53-1 mutants, normalized to the value at t = 0 in wildtype cells. D) Quantification is shown from experiment in Fig 3B comparing half-life of Ndd1 in rgt1Δ and rgt1Δgrr1Δ mutants, normalized to the value at t = 0 in rgt1Δ cells. Small inset on right shows the quantification from darker exposure of rgt1Δ strain only. E) Quantification is shown from experiment in Fig 3D, comparing G1 half-life of Ndd1 in rgt1Δ and rgt1Δgrr1Δ mutants, normalized to the value at t = 0 in rgt1Δ cells. F) Quantification is shown from experiment in Fig 3E, comparing half-life of Ndd1 in wildtype and cdh1Δ mutants, normalized to the value at t = 0 in wildtype cells. G) Quantification is shown from Fig 4D, comparing half-life of Ndd1 in wildtype and mec1Δtel1Δ mutants, normalized to the value at t = 0 in wildtype cells. H) Quantification is shown from Fig 4E, comparing half-life of Ndd1 and epistasis in wildtype, swe1Δ mutants, and mec1Δtel1Δ mutants, normalized to the value at t = 0 in sml1Δ cells. I) Quantification is shown from Fig 4F, comparing half-life of Ndd1 in wildtype and rad53Δ mutants, normalized to the value at t = 0 in wildtype cells. J) Quantification is shown from Fig 4G, comparing half-life of Ndd1wt and Ndd1m27, normalized to the value at t = 0 in Ndd1wt. (TIFF) [file pgen.1005162.s002.tiff]
